# Correlation of brachycephaly grade with level of exophthalmos, reduced airway passages and degree of dental malalignment’ in Persian cats

**DOI:** 10.1371/journal.pone.0254420

**Published:** 2021-07-21

**Authors:** Jana Sieslack, Daniela Farke, Klaus Failing, Martin Kramer, Martin J. Schmidt

**Affiliations:** 1 Department of Veterinary Clinical Sciences, Clinic for Small Animals, Neurosurgery, Neuroradiology and Clinical Neurology, Justus-Liebig University-Giessen, Giessen, Germany; 2 Unit for Biomathematics and Data Processing, Faculty of Veterinary Medicine, Justus-Liebig University, Giessen, Germany; 3 Department of Veterinary Clinical Sciences, Clinic for Small Animals, Surgery, Justus-Liebig University-Giessen, Giessen, Germany; Faculty of Animal Sciences and Food Engineering, University of São Paulo, BRAZIL

## Abstract

For many years, there has been a trend to breed cats with an increasing degree of brachycephalic head features, which are known to have a severe impact on the animals’ health and welfare. The direct relation between different grades of brachycephaly and their negative implications have not been researched in this species. The aim of this study was therefore to establish correlations between the different grades of brachycephaly and reduced upper respiratory airways, exophthalmos of the eye globes and malalignment of the teeth in Persian cats. Sixty-nine Persian cats of various skull dimensions and ten Domestic shorthair cats were recruited for the study. The cats’ skulls were examined using three-dimensional reconstructions created from Computed Tomography datasets. Brachycephaly was graded using established craniometric measurements (facial index, cranial index, skull index, craniofacial angle). The flow area of the nasal passageways at different locations, the amount of the eye globe not supported by the bony orbit and the axial deviation of the teeth were quantified and evaluated for a correlation with the grade of brachycephaly. The results of this study clearly show that increased grades of brachycephaly in Persian cats resulted in larger extra-orbital parts of the ocular bulbs. The brachycephalic skull dimension also resulted in a lower height of the naso-osseal aperture, while other areas of the nasal airways were not correlated with the severity of brachycephaly. Persian cats showed a significantly increased occurrence of premolar tooth displacement in the upper jaw with increasing brachycephaly grades. It was interesting to note that the measured values had a broad range and values of some individual Persian cats showed an overlap with those of Domestic shorthair cats.

## Introduction

For many years, there has been a trend to breed companion animals with an increasing degree of brachycephalic head features [1, 2]. The brachycephalic skull type has also become very popular in cats. Since the middle of the last century, the morphology of the original Persian cat has gradually modified into an extremely flat-faced type with a large prominent forehead, severely reduced facial bones and large, round eyes [2–4]. The original type was largely replaced by extremely brachycephalic cats that define the breed standard of the modern Persian cat today, even though it is known from dogs that high grades of brachycephaly can be associated with severe health problems [2, 5–7]. Research in this field is largely focused on dogs, but there is increasing evidence that brachycephalic cats experience similar health issues to many brachycephalic dog breeds [2, 5–10]. Anatomical changes in the upper respiratory tract such as stenotic nares, elongation of the soft palate or nasopharyngeal turbinates are congenital abnormalities that cause or contribute to upper airway obstruction resulting in breathing abnormalities (brachycephalic obstructive airways syndrome; BOAS) [5]. Due to the reduction of the maxillary alveolar space in modern Persian cats, their teeth are positioned at abnormal angles and overlap each other, causing dental and gingival problems [3, 7, 10]. Modern Persians also have shallow orbits [9] with prominent ocular globes [11], giving these breeds the desired large expressive eyes [4]. These protruding eyes are prone to exposure keratitis and probably contribute to corneal sequestrum development [12–15]. Due to severe distortion of the nasolacrimal duct, tear fluid cannot drain normally from the eyes, explaining the tendency for epiphora [6]. Reduction of maxilla length can cause excessive skin folds on the face, which further predisposes to skin infections, and may contribute to the development of idiopathic facial dermatitis [7]. Furthermore, modern Persian cats have a general propensity for dermatological conditions caused by an inability to groom normally [7]. Finally, it was recently shown that selection towards reduced facial bones also leads to a fundamental change in morphology of the cranial cavity, causing intracranial overcrowding, herniation of the brain and internal hydrocephalus [10].

Veterinary societies in many countries try to improve the health, well-being and welfare of brachycephalic dogs and cats [16–19]. Strategies range from campaigns aiming to improve owner awareness of breed associated diseases [20, 21], promotion of a need for change in breed standards, to the total ban of showing or breeding extreme brachycephalic breeds in a country [22]. Breeding out brachycephaly by the introduction of mesocephalic cats would likely permit the correction of the extreme brachycephalic phenotype. Although this may be an expediate measure, it is less desirable for a breeder as it would change the phenotype, behaviour and characteristics of the cats in many ways. Furthermore, although first generation offspring (F1-generation) of brachy- and mesocephalic cats may share the same desired phenotype, genetic information will most likely segregate in the offspring of F1-matings and following generations [23]. The restoration of less brachycephalic Persians must therefore rely on intra-breed selection. However, there is neither information on the phenotypic variability regarding brachycephalic traits in the Persian cat in Germany There is also no r data showing a clear correlation between increased grades of brachycephaly and adverse morphological changes of the skull, which may have a negative impact on the cats’ health and welfare. We therefore investigated the skull morphology in Persians of different German breeding lines. The aim of the study was to collect data concerning the extent to which brachycephalic head morphology in Persian cats is correlated with exophthalmos, reduced nasal airways and dental malalignment.

## Materials and methods

### Animals

In this prospective cross-sectional study, 69 Persian cats from breeding clubs in Germany were invited for prospective computed tomography (CT) head examinations. There was no criteria defined for the cats to be included into the study. Furthermore, CT studies of ten Domestic shorthair cats (DSH) were collected from the imaging archive of the Clinic for Small Animals, Justus-Liebig University to compare skull morphology. The cats underwent CT for the diagnosis of external,- and internal ear disease. Only cats with an otherwise normal skull morphology were included into the study.

Of the 69 Persian cats, there were 38 males and 31 females, while in the population of DSH were three male and seven female cats. The age of the Persian cats varied between four months and 16 years (mean 4.52 years ± 4.69 years). The age of the DSH ranged between eight months and 15 years (mean 7.77 years ± 4.57 years). The body weight of Persian cats varied between 1.4–5.6 kg (mean 3.05 kg ± 0.85 kg), while the body weight of DSH was between 3.5–6.0 kg (mean 4.7 kg ± 0.85 kg).

### Clinical examination

The Persian cats underwent a general clinical examination with special regard to the presence (yes/no) of respiratory noise (snorting/wheezing), which was assessed in the resting animal. The cats were also examined for ocular discharge and tear staining. Inspection of the oral cavity was conducted under general anaesthesia. Evaluation of the integrity of the corneal epithelium was determined using a fluorescein test, also under general anaesthesia.

### Ethics statement

This prospective study was carried out in strict accordance with the recommendations in the Guidelines for Care and Use of Laboratory Animals of the German Animal Protection Law. The protocol was approved by the Committee on the Ethics of Animal Experiments of the Justus-Liebig University-Giessen and the government of the (Regierungspräsidium) Hessian State (Permit number: 560 AZ- Gi 18/17 Nr. A20/2013). All cats lived with their owners, who gave permission for their animals to be used in this study.

### Anaesthesia

CTs were performed under general anaesthesia to exclude motion artefacts. Anaesthesia was induced with diazepam (0.5 mg/kg IV; Diazepam-ratiopharm, ratiopharm GmbH, Ulm, Germany) and propofol (2–4 mg/kg IV; Vetofol, Bayer Vital GmbH, Leverkusen, Germany), and maintained by isoflurane (1.5–2.0%; Isofluran CP, cp-pharma GmbH, Burgdorf, Germany) after intubation.

### Image acquisition

#### Computed tomography (CT)

The CT data sets were acquired with a 16-slice helical CT scanner (CT Brilliance, Philips, Hamburg, Germany; 120 kV, 350 mAs, matrix 512 x 512, slice thickness 0.8 mm, pitch factor 1). The cats were positioned in sternal recumbency with the head slightly elevated.

#### Image processing

The skulls of the cats were examined using 3D models created from the CT datasets. Image processing for volume rendering of the volumes of interest was achieved using specialised graphical software (AMIRA^®^, Mercury Computers Systems, Berlin, Germany). This program combines image information of two or more different planes in association with three-dimensional (3D) models, which allows accurate manual and semi-automatic image segmentation on a slice-by-slice basis. The skulls of the cats were semi-automatically reconstructed and 3D computerised models were generated from the two-dimensional (2D) CT images using a threshold that enabled visualisation of bone. The 3D models were combined with orthogonal 2D CT images (ortho-slice) and reconstructed image planes (oblique slice), allowing definite identification of measuring points.

#### Craniometric analysis

**Fig 1:**
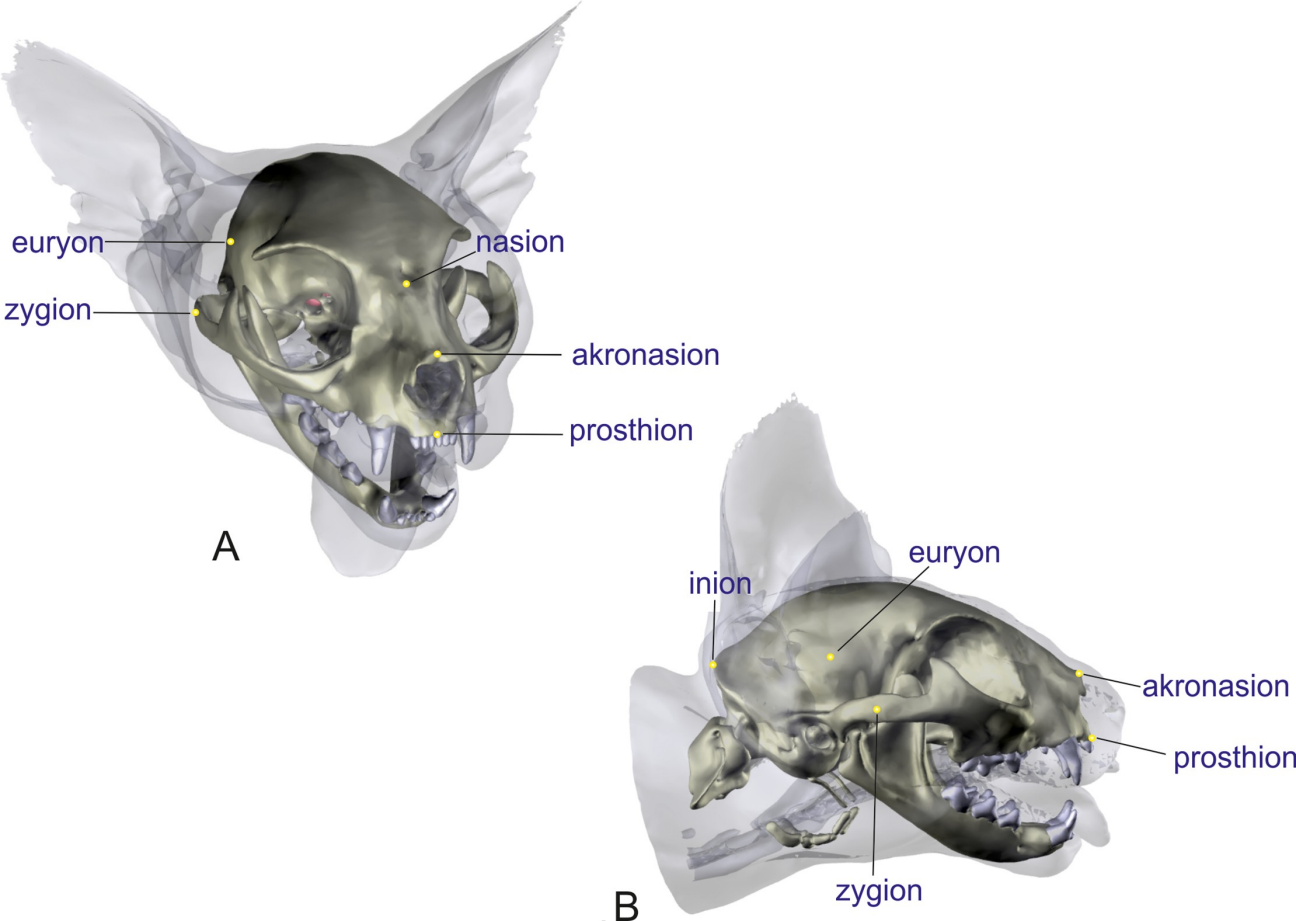
Craniometric measuring points on the skull of a Domestic shorthair cat. Three-dimensional reconstructions of a Domestic shorthair cat with a low grade of brachycephaly in a frontolateral view (A) and lateral view (B) demonstrating the craniometric landmarks. Linear measurements were obtained between the craniometric landmarks.

Measuring points that were not clearly seen in the models (for instance the naso-frontal suture) were identified and set in a 2D-ortho-slice combined with the model. The landmarks for craniometric measurements were previously defined for dry skulls and radiographs [24, 25] and were previously used for analysis of the 3D models based on the CT datasets [26]. For each group, the following definitions were applied:

**Inion**: Central surface point on the external occipital protuberance (Fig 1A).

**Nasion**: Junction on the medial plane of the left and right naso-frontal sutures (Fig 1A).

**Akronasion**: Most rostral end of the nasal bone measured in the same plane as the nasion (Fig 1A and 1B).

**Prosthion**: Rostral end of the interincisive suture, located between the roots of the superior central incisor teeth (Fig 1A and 1B).

**Zygion**: The most lateral point on the zygomatic arch (Fig 1A and 1B).

**Euryon**: The most lateral part of the outside of the braincase on either parietal bone (Fig 1A and 1B).

**Facial length**: Distance from nasion to prosthion.

**Facial width**: Distance between left and right zygion.

**Cranial length**: Distance from inion to nasion.

**Cranial width**: Distance between left and right euryon.

**Skull length**: Distance from inion to prosthion.

**Skull width**: Distance between the left and right zygion.

**Facial index**: Ratio of the facial width to the facial length. A high facial index indicates a wider facial skull in relation to length (i.e. a higher grade of brachycephaly).

**Cranial index**: Ratio of the cranial width to the cranial length. A high cranial index indicates a wider braincase in relation to length (i.e. a higher grade of brachycephaly).

**Skull index**: Ratio of the skull width to the skull length. A high skull index indicates a wider skull in relation to length (i.e. a higher grade of brachycephaly).

**Fig 2:**
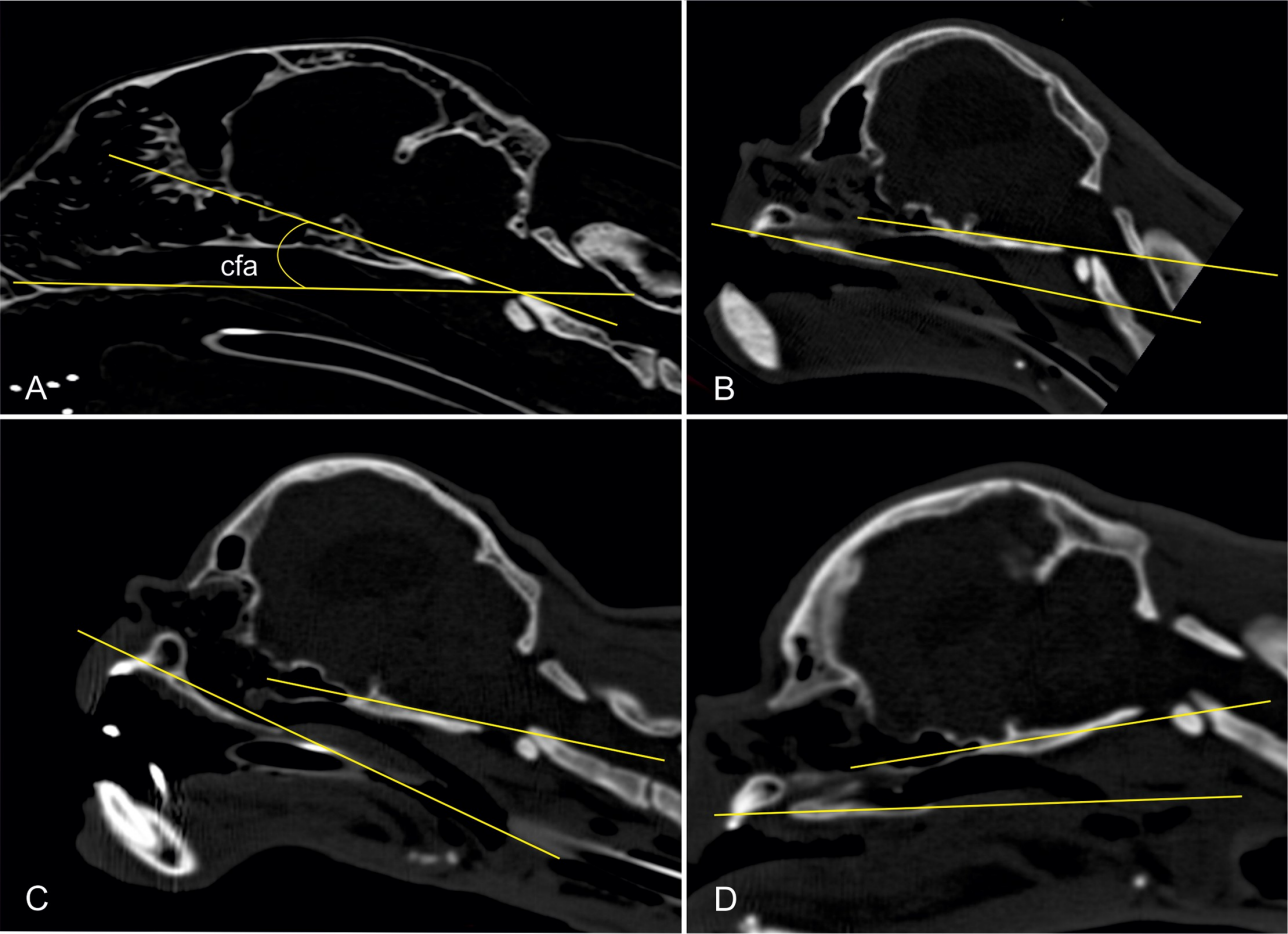
Measurement of the craniofacial angle. Two-dimensional CT images in a sagittal plane of a Domestic shorthair cat (A) and Persian cats with different cranial indices (B–D). The craniofacial angle is determined between the facial axis and the basilar axis. In Fig 2B–2D, the craniofacial angle (cfa) cannot be measured because the two axes diverge.

Furthermore, the craniofacial angle between the basilar and facial axes [24] was determined in 2D CT images. The basilar axis runs through the caudal border of the chiasmatic sulcus to the basioccipital bone and is extended caudally, while the facial axis represents the extension of the hard palate (Fig 2). A small craniofacial angle implies a higher grade of brachycephaly.

### Volume and area measurements

#### Extra-orbital parts of the eyes

**Fig 3:**
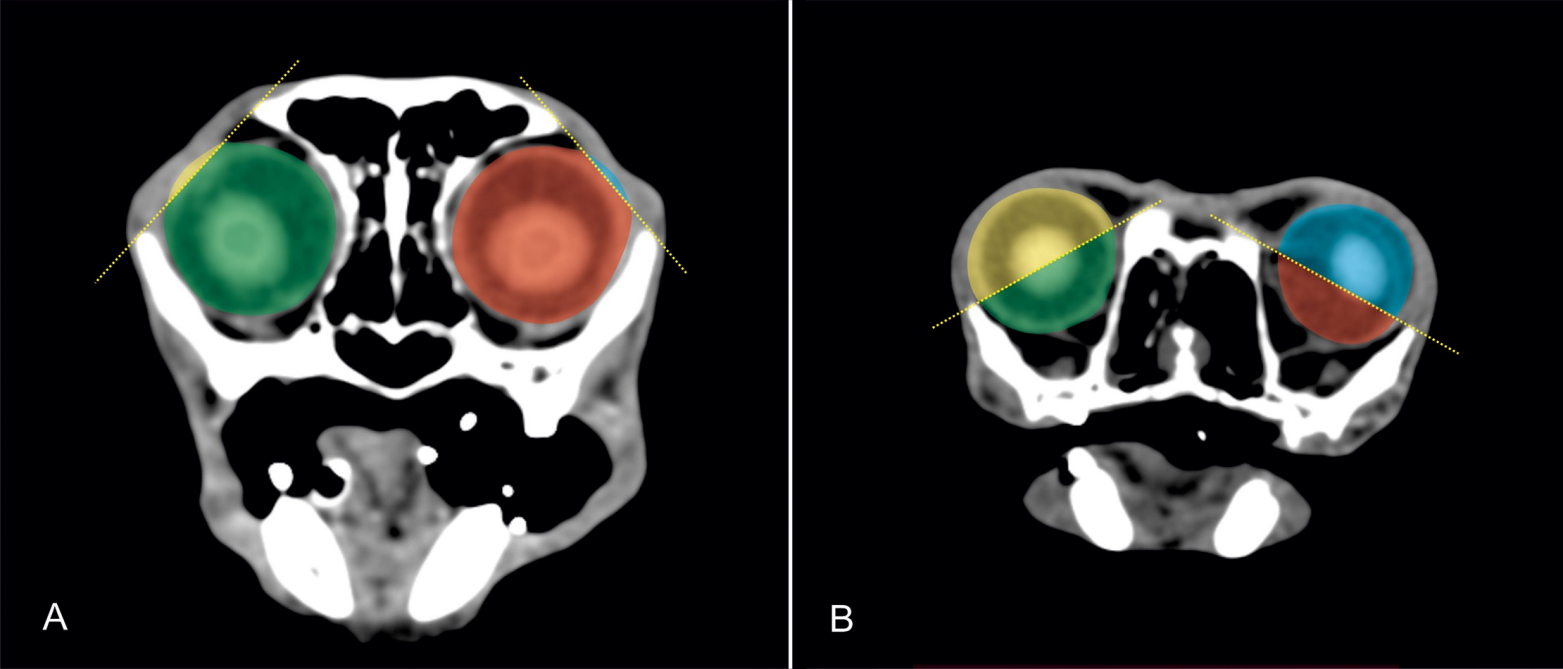
Measurement of the extra-orbital parts of the eye globes. Two-dimensional CT images in a transversal plane of a Domestic shorthair cat (A) and a Persian cat (B) demonstrating the segmentation of the intra-orbital and extra-orbital parts of the bulbi oculi: yellow = extra-orbital part of the right eye; green = intra-orbital part of the right eye; red = intra-orbital part of the left eye; blue = extra-orbital part of the left eye.

The volume of the ocular globes was determined based on the CT datasets using a threshold that enabled visualisation of soft tissue. Ocular muscles were not included. In order to separate the intra- and extra-orbital parts of the globe, the supraorbital (margo supraorbitalis) and infraorbital margin (margo infraorbitalis) of the 3D model were marked using the ‘landmark tool‘. An ‘oblique slice’ was set through these landmarks that appeared as a red line in every image plane reconstructed by the software. This line marked the border between intra- and extra-orbital parts. During the segmentation process, the parts of the globe on either side of the oblique slice were automatically blocked from segmentation to get a clearly defined border between the globe parts. The volume of the extra-orbital and intra-orbital part of the ocular globe were manually segmented by tracking the outlines of the globe (Fig 3). The volumes of the extra-orbital and intra-orbital parts of the eyes could be calculated using the function ‘material statistics’. The percentage of the extra-orbital globe in relation to the entire volume of the globe was calculated.

#### Measurements of the nasal airways

**Fig 4:**
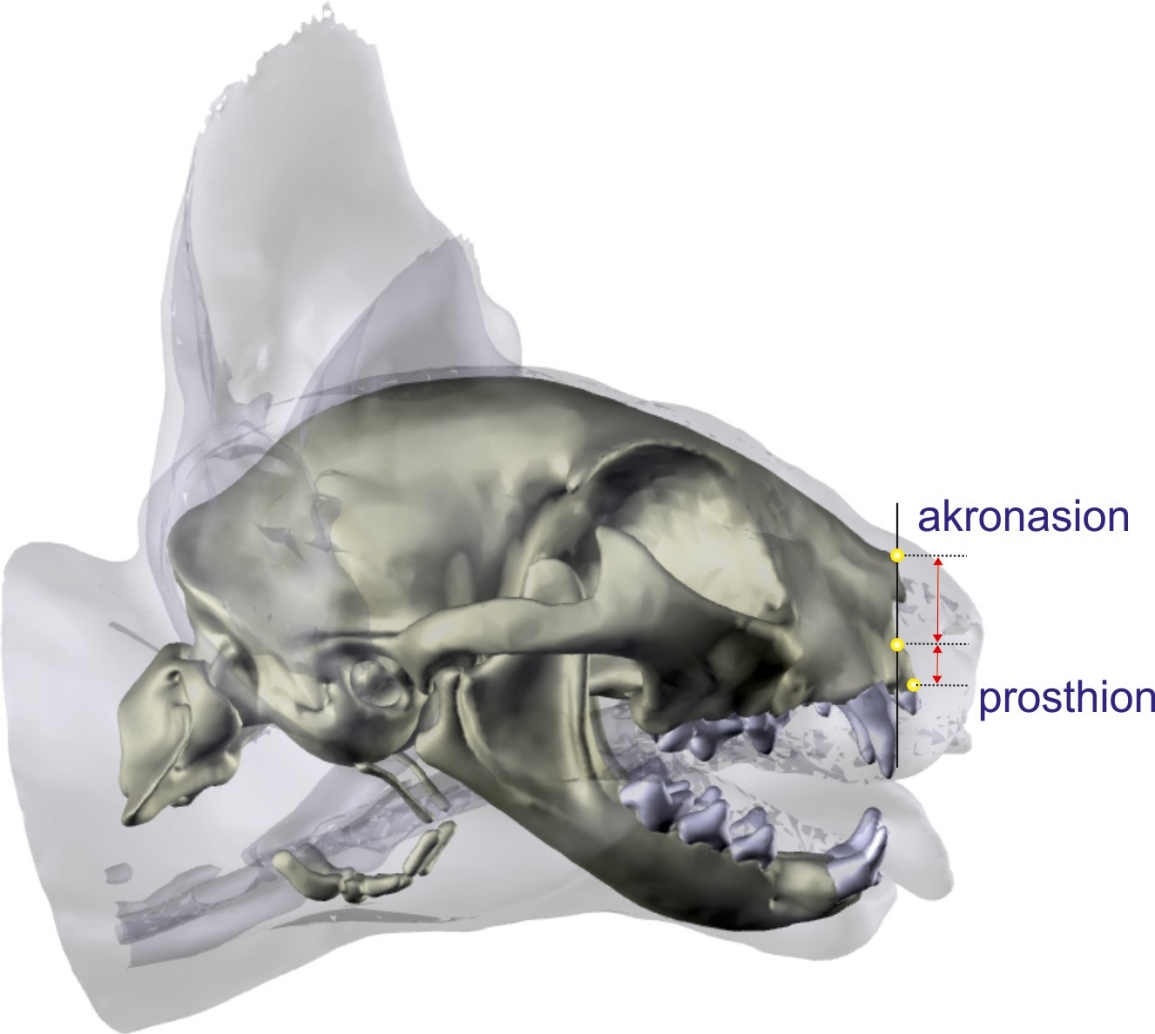
Measurements of the relative height of the bony nasal aperture. Three-dimensional reconstructions of a Domestic shorthair cat in a lateral view. The distance from the akronasion to the midpoint of the palatine process of the incisive bone compared with the distance from the prosthion to that point in the same plane to determine the relative height of the bony nasal aperture.

First, the relative height of the osseous nasal aperture was determined in 3D skull reconstructions. A line between the akronasion and the junction of the palatine process of the incisive bone with the palatine bone served as a first reference line. In the same alignment, a second measurement from the akronasion to the ventral margin of the nasal aperture was done (Fig 4). These two measurements were compared to determine the relative height of the bony nasal aperture. Second, the relative area of the left and right airway passages was evaluated in seven transversal planes based on 2D CT images. In a first step, the total area of the inner nasal cavity including conchal bones, septum and mucosal tissue were measured in defined transverse sections. Then, the air-filled area of this plane was segmented using semi-automated segmentation tools based on the Hounsfield units of air and compared to the total area. Left and right airway passages and nasal cavities were calculated together.

**Fig 5:**
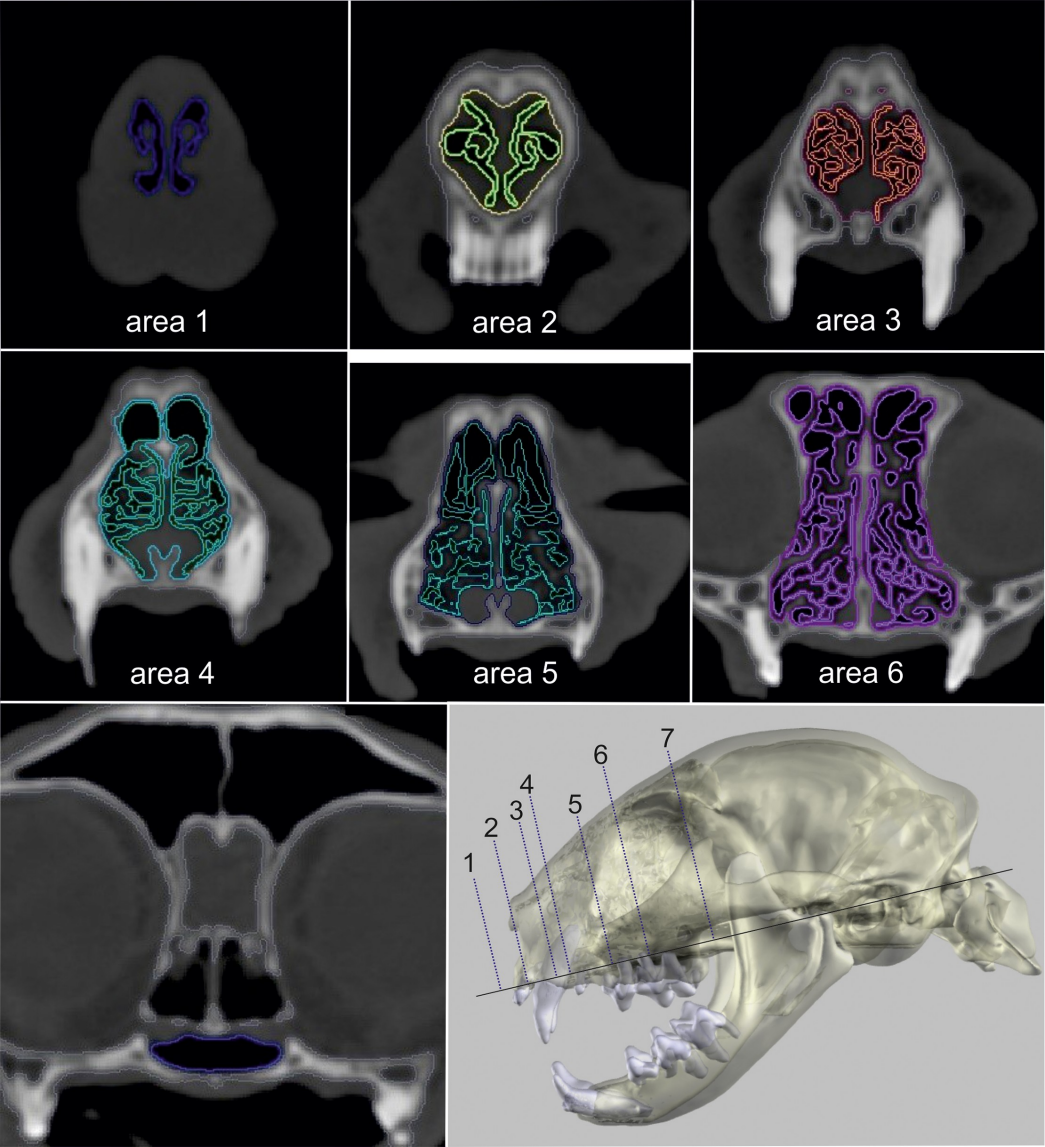
Measurement of nasal airway passage in a Domestic shorthair cat. Two-dimensional CT images in a transversal plane of the nasal cavity in a Domestic shorthair cat with segmentation of the airway passages and the surrounding tissues.

**Fig 6:**
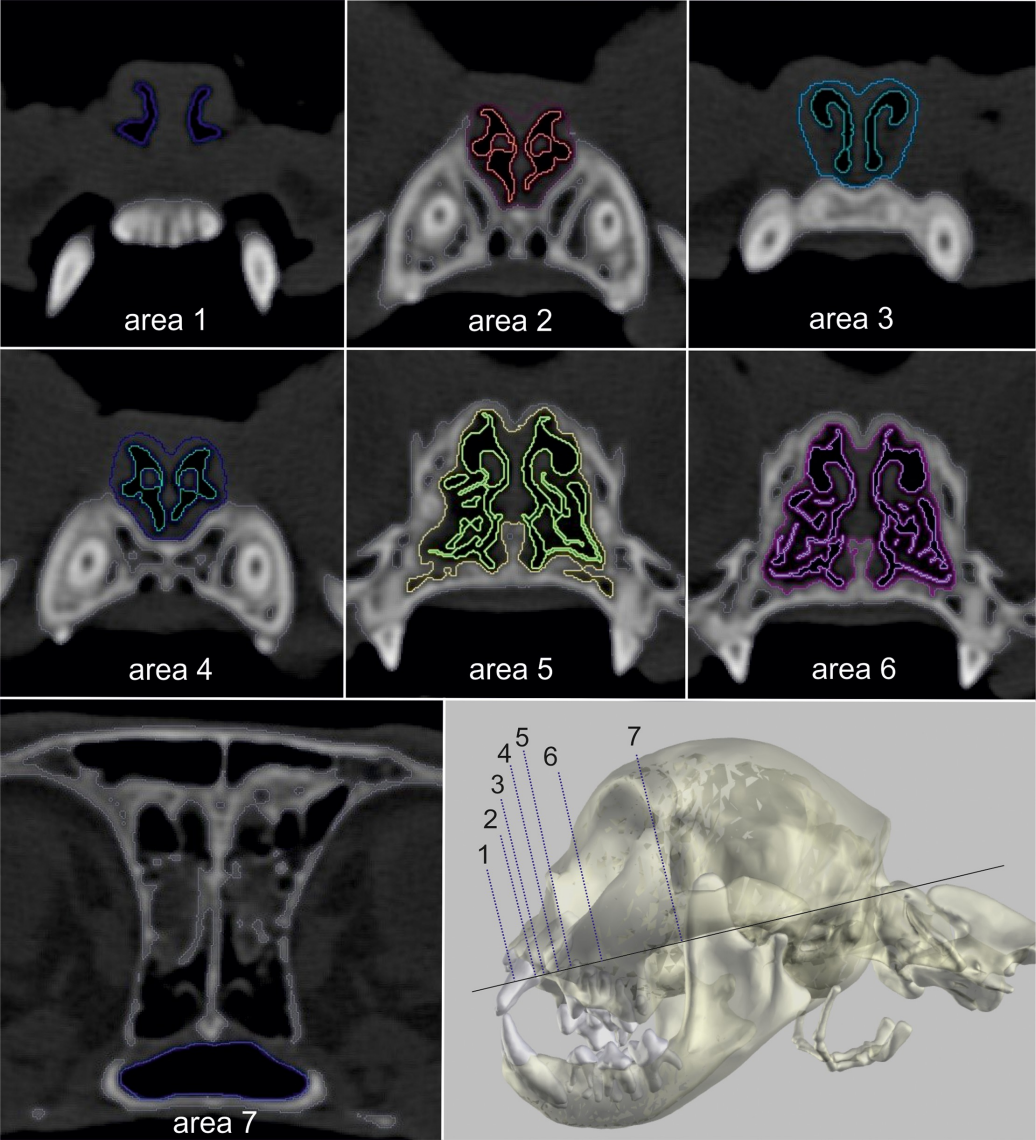
Measurement of the nasal airway passages in a Persian cat. Two-dimensional CT images in a transversal plane of the nasal cavity in a Persian cat with segmentation of the airway passages and the surrounding tissues.

The following cross-sectional areas were examined in a transversal plane:

**Nostrils (area 1)**: The area of the (nasal openings) nostrils was measured in a plane, in which the dorsal, ventral and accessory nasal cartilage formed a continuous outline (Figs 5 and 6).

**Apertura nasi ossea (area 2)**: Nasal airways at the level of the akronasion (Figs 5 and 6).

**Canine tooth (area 3)**: Nasal airways were measured at the level of the midpoint of the canine tooth (Figs 5 and 6).

**Fissura palatina (area 4)**: Nasal airways at the level immediately before the breakthrough of the palatine fissure by the incisive bone (Figs 5 and 6).

**P2 (area 5)**: Nasal airways at the level where the maximum size of the second upper (maxillary) premolar was visible (Figs 5 and 6).

**P3 (area 6)**: Nasal airways at the level of the main tubercle of the third maxillary premolar (Figs 5 and 6).

**Fig 7:**
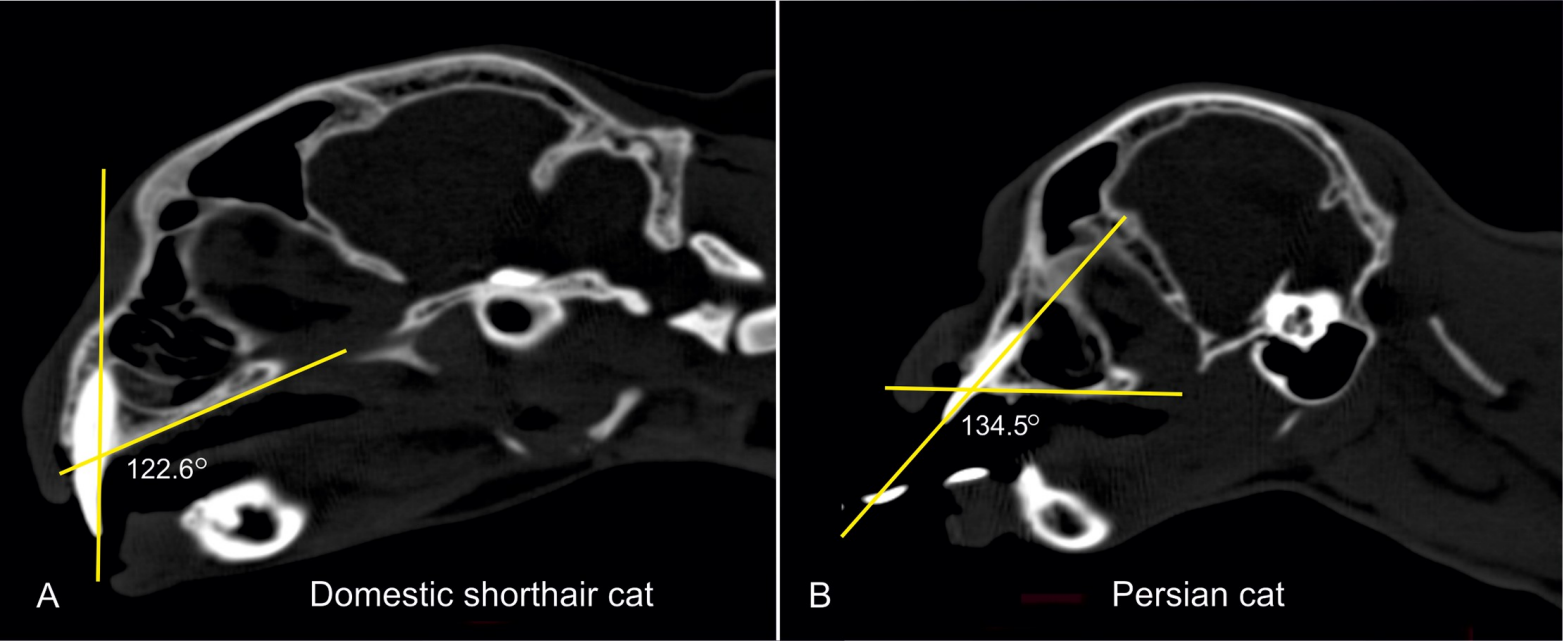
Measurement of the angle between the canine tooth and the hard palate. Two-dimensional CT images in a sagittal plane of an less severebrachycephalic Persian (A) and an extreme brachycephalic Persian (B) demonstrating the measurement of the angle between the canine tooth and hard palate. Axis (1) passes through the dental root and the dental crown. Axis (2) runs along the most cranial and caudal points of the hard palate. Both axes are coloured yellow. Ventrally, between the two axes, the angle is measured in degrees.

**Fig 8:**
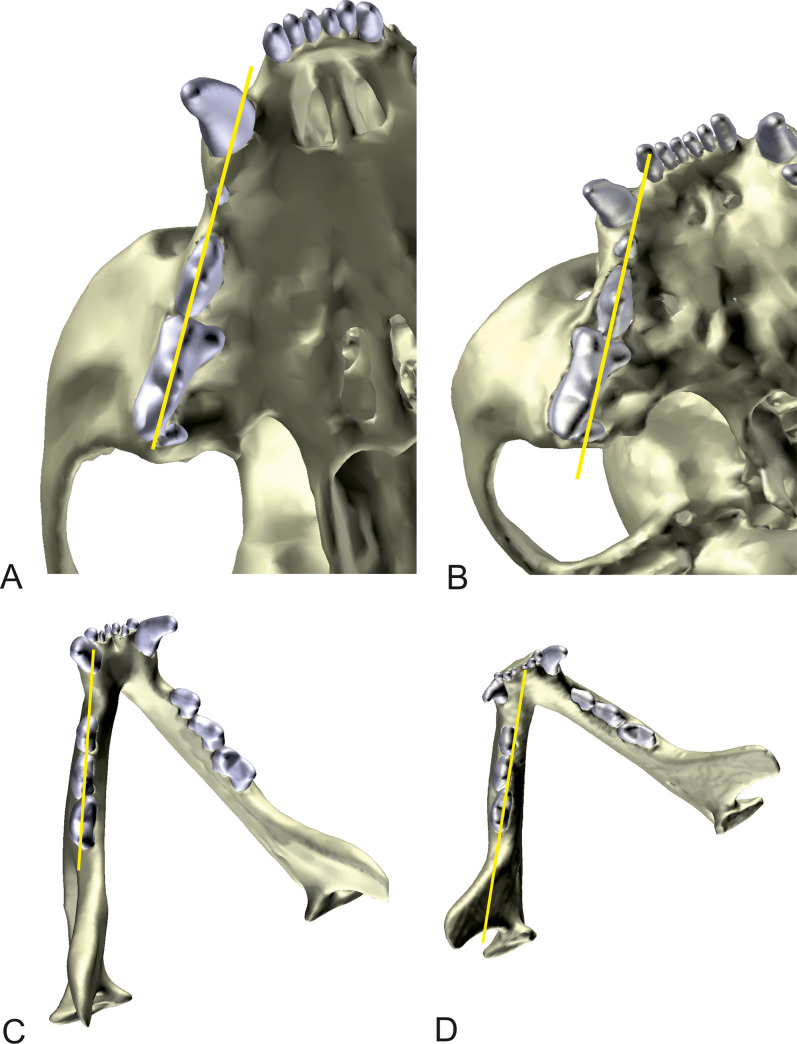
Measurement of dental malalignment in the maxilla and mandibula. Three-dimensional reconstructions of the skull and teeth in a Domestic shorthair cat (A) and a Persian cat (B). The mandibula is removed from the model to assess the maxilla. Auxiliary lines (yellow) through the main tubercles of the second and third premolars of the upper and lower jaws were set, which represent the main axis of the alveolar spaces. If one or more tubercles of the following premolars were positioned laterally to the auxiliary line, the position of the tooth was considered incorrect.

**Relative area of the meatus nasopharyngeus (area 1/7)**: Airways in the sinus were excluded (Fig 5). The air-filled area of the nares was divided by the cross-sectional area of the meatus nasopharyngeus to compare the beginning and the end of the nasal cavity. Correct localisation of the planes was verified in sagittal sections (Fig 7) and in the 3D model (Fig 8).

### Measurement of dental malalignment

#### Angle between canine teeth and hard palate

To determine the angle between the canine teeth and the hard palate in DSHs and Persians, the sagittal plane was used in the 2D model. At the plane of the dental pulp of the canine tooth, two axes were used. One axis passed through the dental root and the dental crown of the canine tooth, while the other axis passed through the most rostral and caudal point of the hard palate. Ventrally, between these two axes, the angle was measured (Fig 7). To emphasise the extreme case of a brachycephalic skull, the maximum angle of both canine teeth was chosen for the evaluation. During this measurement, it was possible to determine whether a dorsal rotation of the maxilla and simultaneously, the hard palate existed.

#### Dental malalignment of the maxilla and the mandibular

Auxiliary lines through the main tubercles of the second and third premolars of the upper and lower jaws were set, which represent the main axis of the alveolar spaces. If one or more tubercles of the following premolars were positioned laterally or medially to the auxiliary line, the position of the tooth was considered incorrect (Fig 8). The lower jaw was examined in the same manner. The number of incorrectly located teeth of the upper and the lower jaws were added separately and included in the evaluation.

### Statistical analysis

Statistical analysis was performed using the commercial statistical software packages Graph Pad Prism and BMDP (BMDP Statistical Software, Inc., Los Angeles, USA). Graphical presentations were created using Graph Pad Prism. First, mean values, standard deviations, maxima and minima of the Persian cat population and the population of DSH regarding the measured variables were determined (BMDP1D program). Subsequently, the data of both populations were checked for normal distribution using the SPSS program (IBM software). For this purpose, the Kolmogorov–Smirnov test and the Shapiro–Wilk test were used. If these results were normally distributed, the Student’s t-test was used. In the t-test, equality of variances using the Levene`s test was considered and the corresponding p-value was considered. If the results were not normally distributed the Mann–Whitney U test was considered. As usual, a significance level of p < 0.05 was used.

The associations between the influencing variables and the target variables were investigated by correlation and regression analysis (BMDP6D program). The correlation coefficients according to Pearson (r) as well as regression lines were analysed. In the examination of the hard palate, a one-factorial logistic regression analysis was performed using the BMDPLR program. A significance level of p < 0.05 was set. Thus, results with p < 0.05 and F > 4.00 were considered statistically significant. Additionally, the odds ratio was determined whether the hard palate occurred in a curved or straight shape in connection with the independent variables.

By means of a multiple linear regression analysis using the BMDP1R program, it was determined which potential influencing factors showed the most statistically significant correlations. Thus, the influence variables sex, age and body weight as a fixed model were analysed for correlations with the target variables in each alternative model with the facial index, the cranial index, the skull index, as well as the craniofacial angle. The investigations of the hard palate were performed by multiple logistic regression analysis (BMDPLR). Logistic regression analysis also allowed the calculation of statistical correlation of alternative models with the facial index, the cranial index, the skull index and the craniofacial angle, each in a fixed model with the influence variables sex, age and body weight.

## Results

### Clinical evaluation

Twenty-one Persian cats had tear staining in both eyes, while 48 cats did not have staining underneath the eyes. The cats with tear staining had significantly higher cranial indices (CI: 90.5 ± 13.29 vs. CI: 79.2 ± 15.04; p = 0.0049) and skull indices (SI: 99.5 ± 7.4 vs. SI 88.83 ± 11.7; p = 0.002). None of the cats had a positive fluorescein test.

Twelve cats had respiratory noise at rest. There was no significant difference in the grades of brachycephaly between cats with or without respiratory noise. Eight Persians had gingivitis and/or ulcerations of the gums. There was no significant difference in the grades of brachycephaly between the cats with or without gingivitis and/or periodontal ulcerations.

### Craniometric parameters

Three of the four craniometric parameters were significantly different between the population of Persian cats and DSH. The measured values are summarised in Table 1. The values indicate a relatively shorter facial skull and a short, wide braincase in Persian cats compared to DSH. However, it is interesting to note that there was an overlap of craniometric indices in Persian cats and DSH.

**Table 1:** Summary of the comparison of the craniometric indices between the Persian and Domestic shorthair cats.

**Breed**	Facial index	Cranial index	Skull index
**Persian cat**	256.03 ± 35.40	85.20 ± 13.93	94.55 ± 9.65
**Domestic shorthair cat**	186.64 ± 11.69	61.48 ± 2.73	72.26 ± 2.27
**p-value**	**P < 0.001**	**P < 0.001**	**P < 0.001**

**Fig 9:**
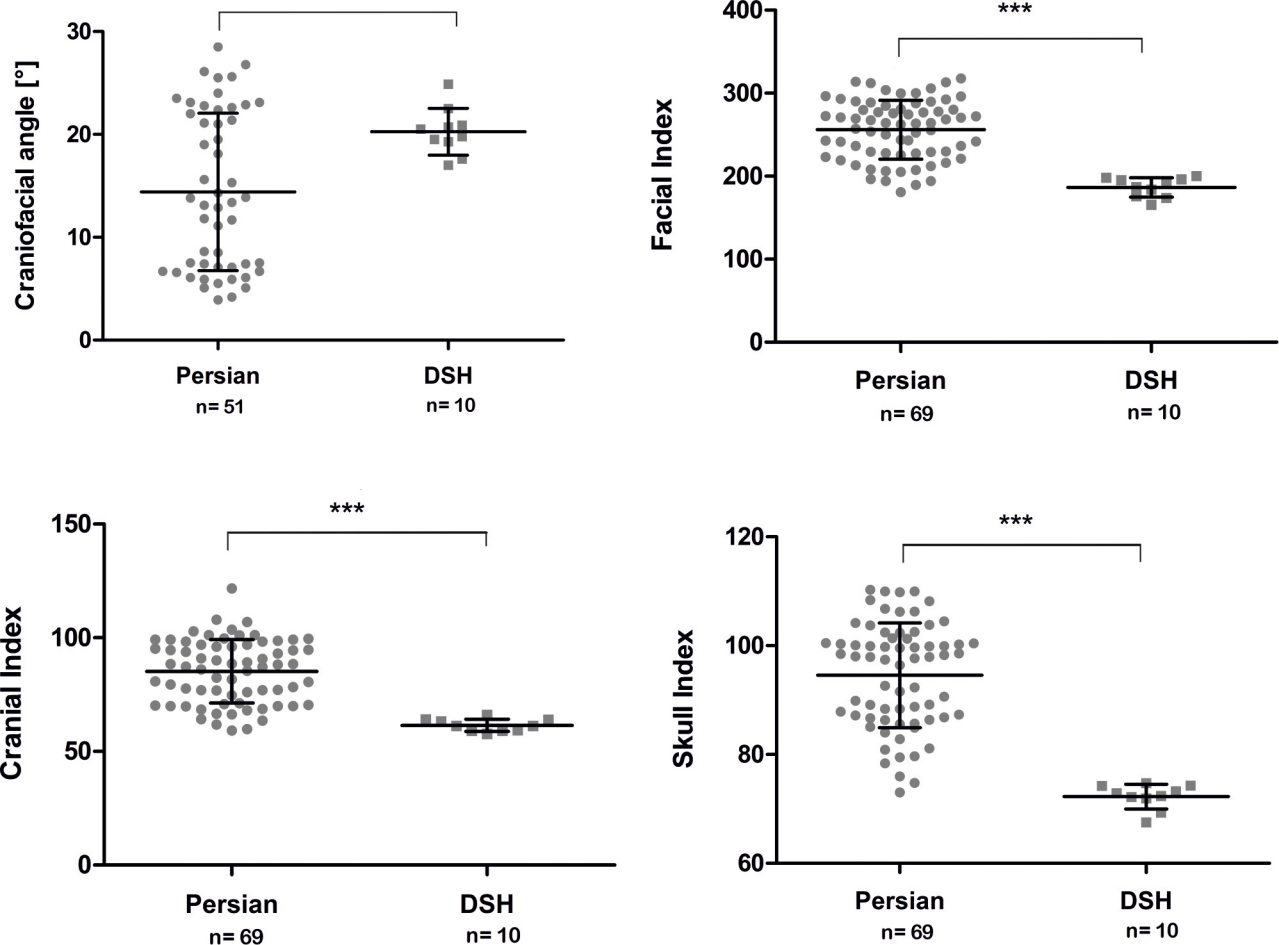
Comparison of craniometric properties between Persian and DSH cats. Graphic representation of the facial index, the cranial index, the skull index and the craniofacial angle, comparing Persian and DSH cats. The values of the individual cats, the mean values, the standard deviations and the significance level are indicated.

Although the brachycephalic skull conformation of Persian cats resulted in smaller craniofacial angles than in cats with mesocephalic skull conformation (Fig 9), there was no significant difference between the mean craniofacial angle of the Persian cats (14.41° ± 7.65°) and the mean craniofacial angle of DSH (20.27° ± 2.28°) (p = 0.066).

### Extra-orbital part of the eyes

**Fig 10:**
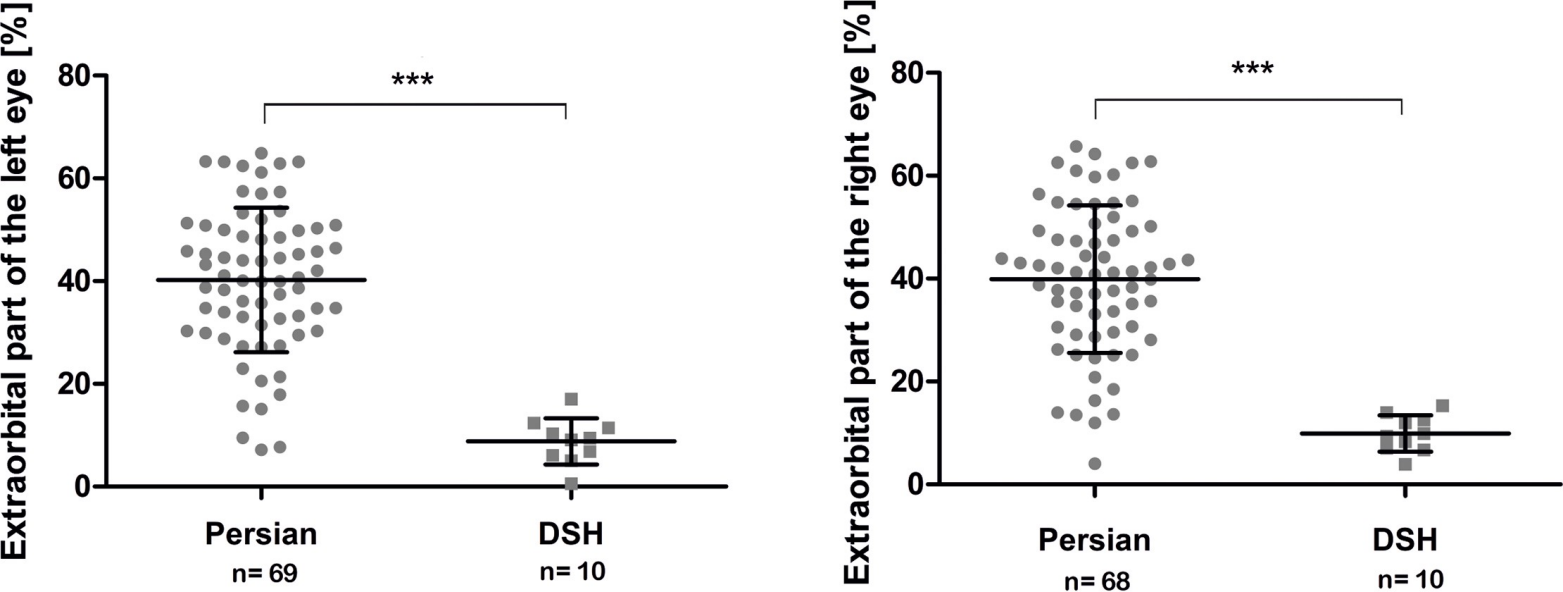
Comparison of the extra-orbital parts of the eye globes between Persian and DSH cats. Graphic representation of the extra-orbital parts of the right and left eye globes, comparing Persian and DSH cats. The values of the individual cats, the mean values, the standard deviations and the significance level are indicated. * = p < 0.01; ** = p < 0.001 *** = p < 0.0001.

The mean extra-orbital part of the ocular globes was significantly higher in Persian (right eye: 39.9% ± 14.32%; left eye: 40.22% ± 14.05%) than in DSH (right eye: 9.90% ± 3.56%; left eye: 8.81% ± 4.51%; p < 0.0001; Fig 10). No significant differences were found between right and left eyes (p > 0.05 each). There was a high variability concerning extra-orbital parts of the bulbi in the Persian group (right eye: 4.06–65.72%; left eye: 7.16–64.87%), whereas the extra-orbital part of the globe in the DSH had a smaller range (right eye: 3.93–15.33%; left eye: 0.54–17%).

**Fig 11:**
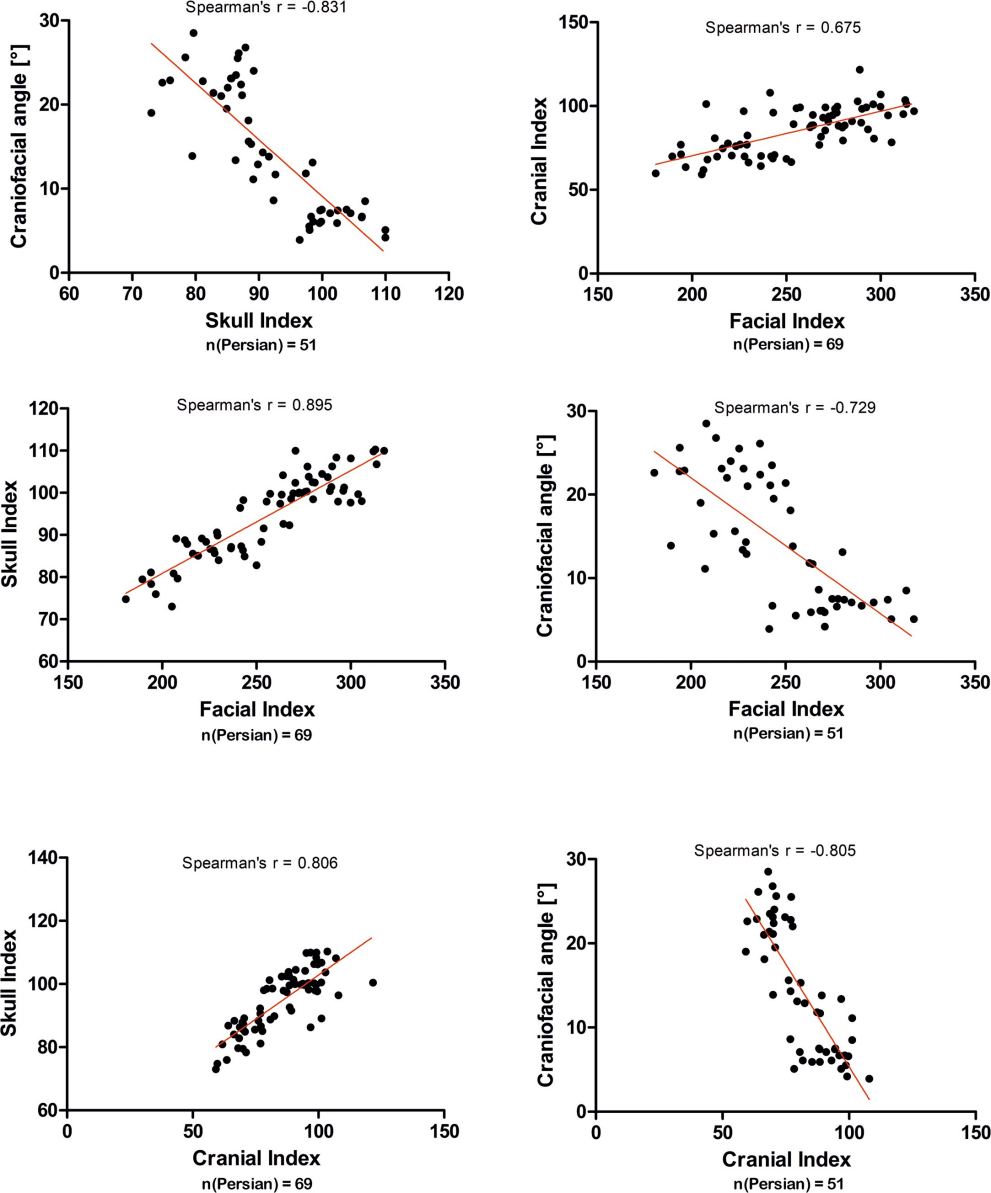
Correlation of measured craniometric parameters with extra-orbital parts of the eyes. Graphic representation of the craniofacial angle, facial index, cranial index, skull index and in correlation with extra-orbital parts of the right eye globes within the Persian cat population (linear regression analysis). The red line represents the regression line. *** = p < 0.0001.

**Fig 12:**
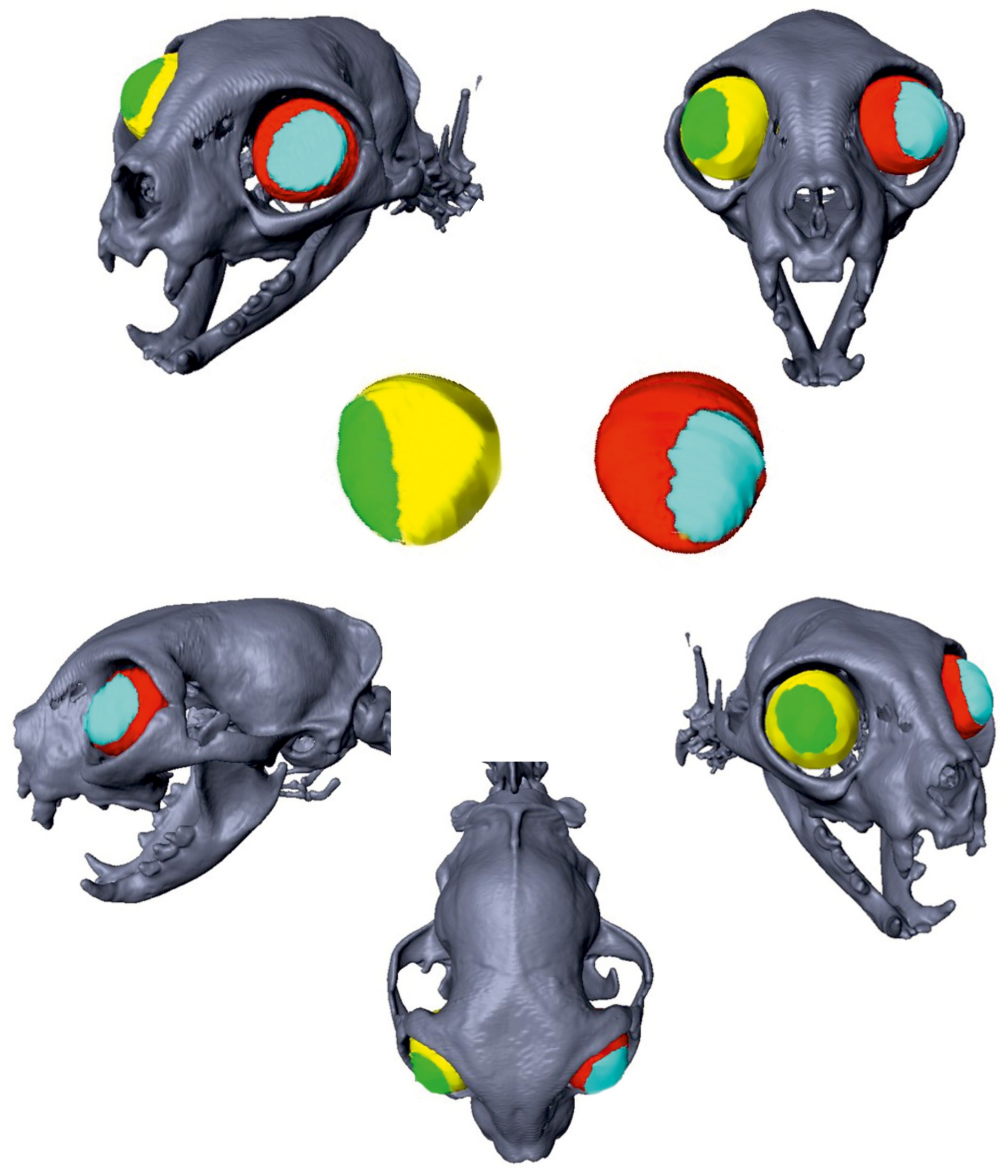
Reconstruction of the extra-orbital part of the eye globes of a Domestic shorthair cat. Three-dimensional skull reconstructions of a Domestic shorthair cat. The different parts of the eye globes are marked in different colours: yellow = intra-orbital part of the right eye; green = extra-orbital part of the right eye; red = intra-orbital part of the left eye; blue = extra-orbital part of the left eye.

**Fig 13:**
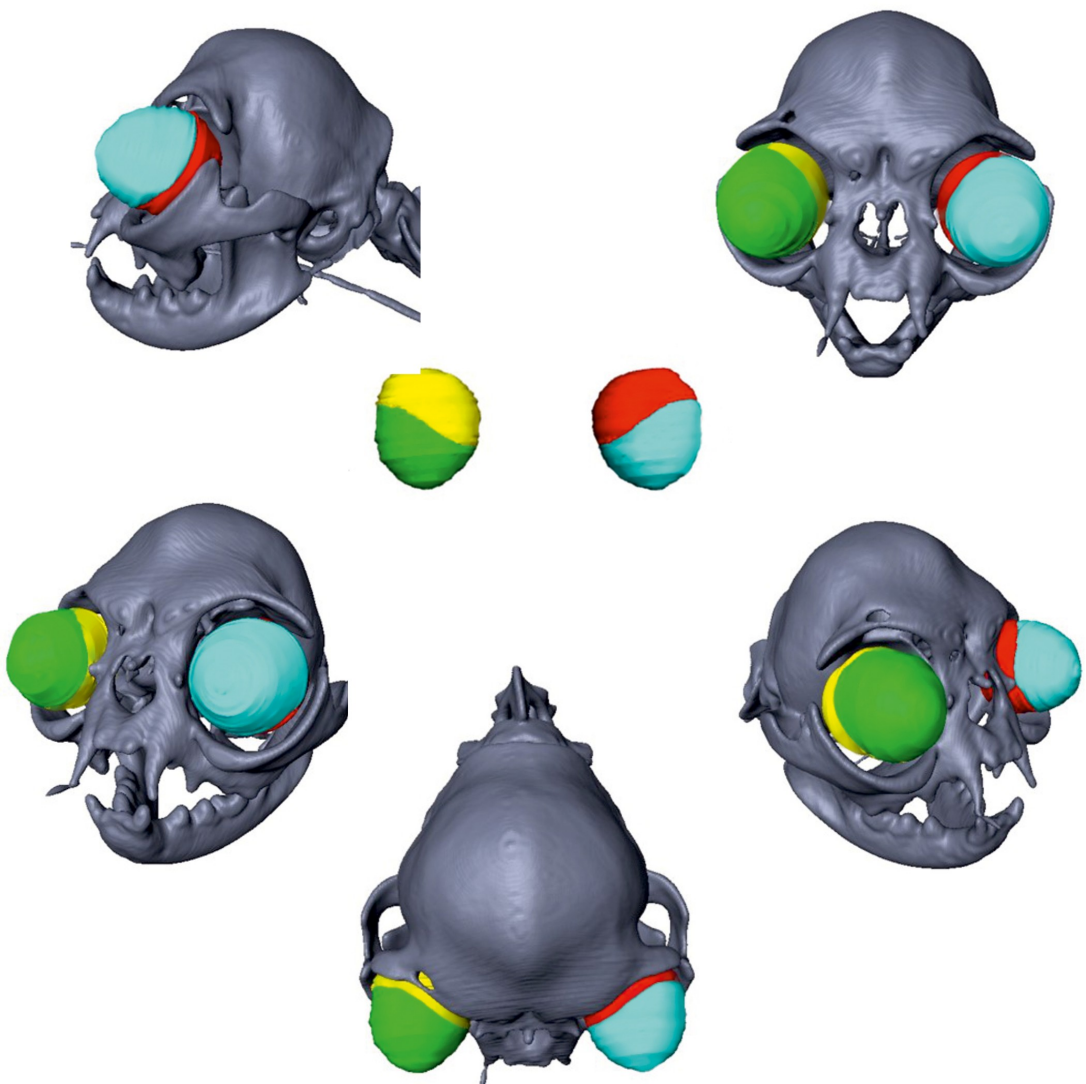
Reconstruction of the extra-orbital part of the eye globes in a Persian cat. Three-dimensional reconstructions of an extreme brachycephalic Persian in the right column. The different parts of the eye globes are marked in different colours: yellow = intra-orbital part of the right eye; green = extra-orbital part of the right eye; red = intra-orbital part of the left eye; blue = extra-orbital part of the left eye. The extremely exposed position of the bulbi in the Persian is evident.

There was a significant correlation between the degree of brachycephaly and the extra-orbital part of the globe within the Persian cat population. Skull index (left eye: p < 0.001, r = 0.508; right eye: p < 0.001, r = 0.536) and cranial index (left eye: p < 0.001, r = 0.608; right eye: p < 0.001, r = 0.557) correlated positively with a larger extra-orbital part of the globe, while the facial index was negatively correlated (left eye: p < 0.001, r = −0.602; right eye: p < 0.001, r = −0.532; Fig 11). In sum, the shorter the facial bones, and the shorter and wider the braincase, the less of the ocular globe was supported by the orbit (Figs 12 and 13).

Multiple linear regression analysis showed that the percentage of the extra-orbital part was most closely related to the skull index (left eye: p < 0.001, F = 26.42; right eye: p < 0.001, F = 21.62) and the cranial index (left eye: p < 0.001, F = 24.6; right eye: p < 0.001, F = 20.16). An increase in the skull index by one unit caused an increase in the extra-orbital part of the right eye by 0.799% and the left eye by 0.804%. Age, sex and body weight had no influence on the location of the eye globes (p > 0.05).

### Nasal airways

**Fig 14:**
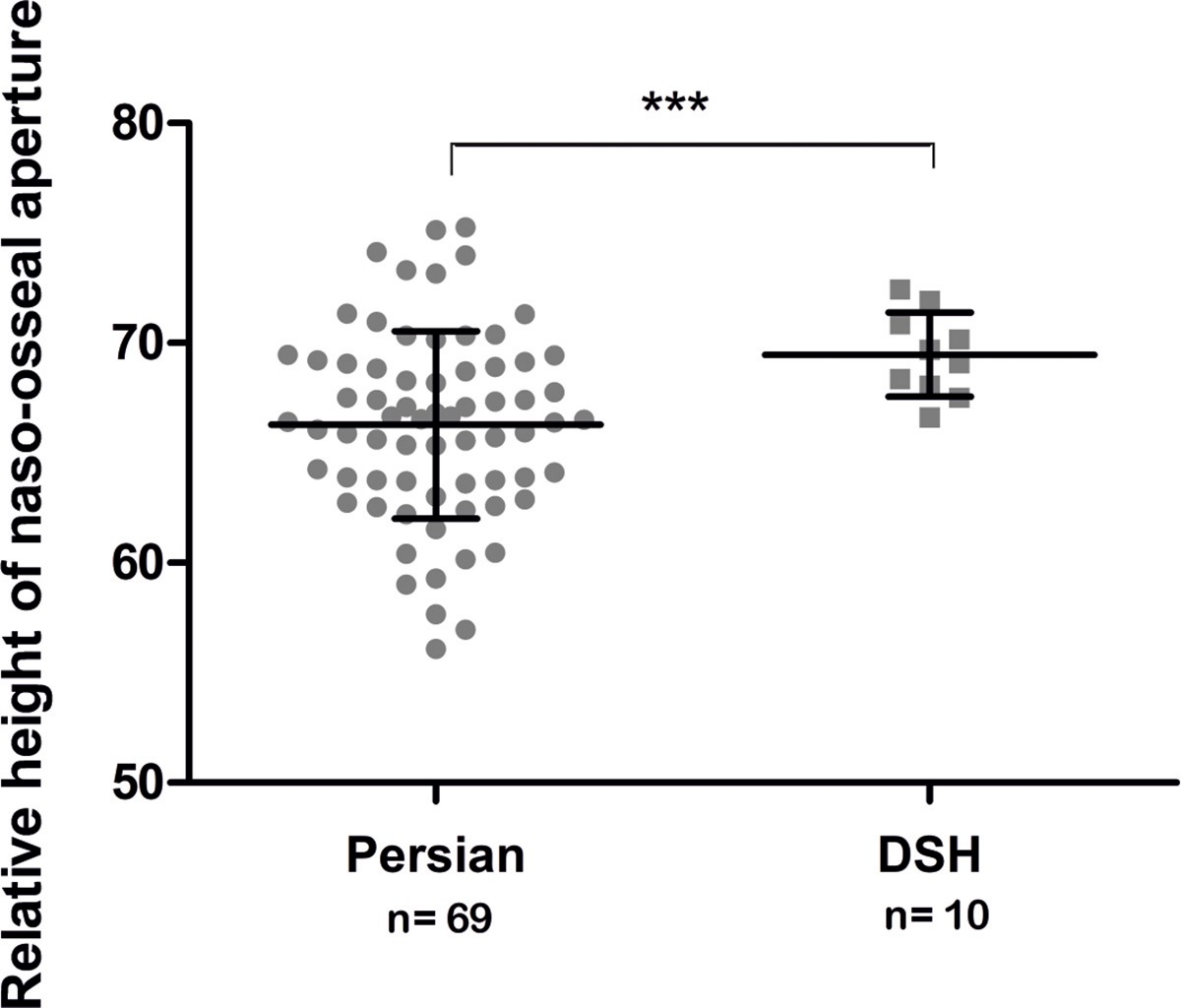
Comparison of the relative height of the naso-osseal aperture between Persians and DSHs. Graphic representation of the relative height of the naso-osseal aperture, comparing Persian and DSH cats. The values of the individual cats, the mean values, the standard deviations and the significance level are indicated. *** = p < 0.0001.

**Fig 15:**
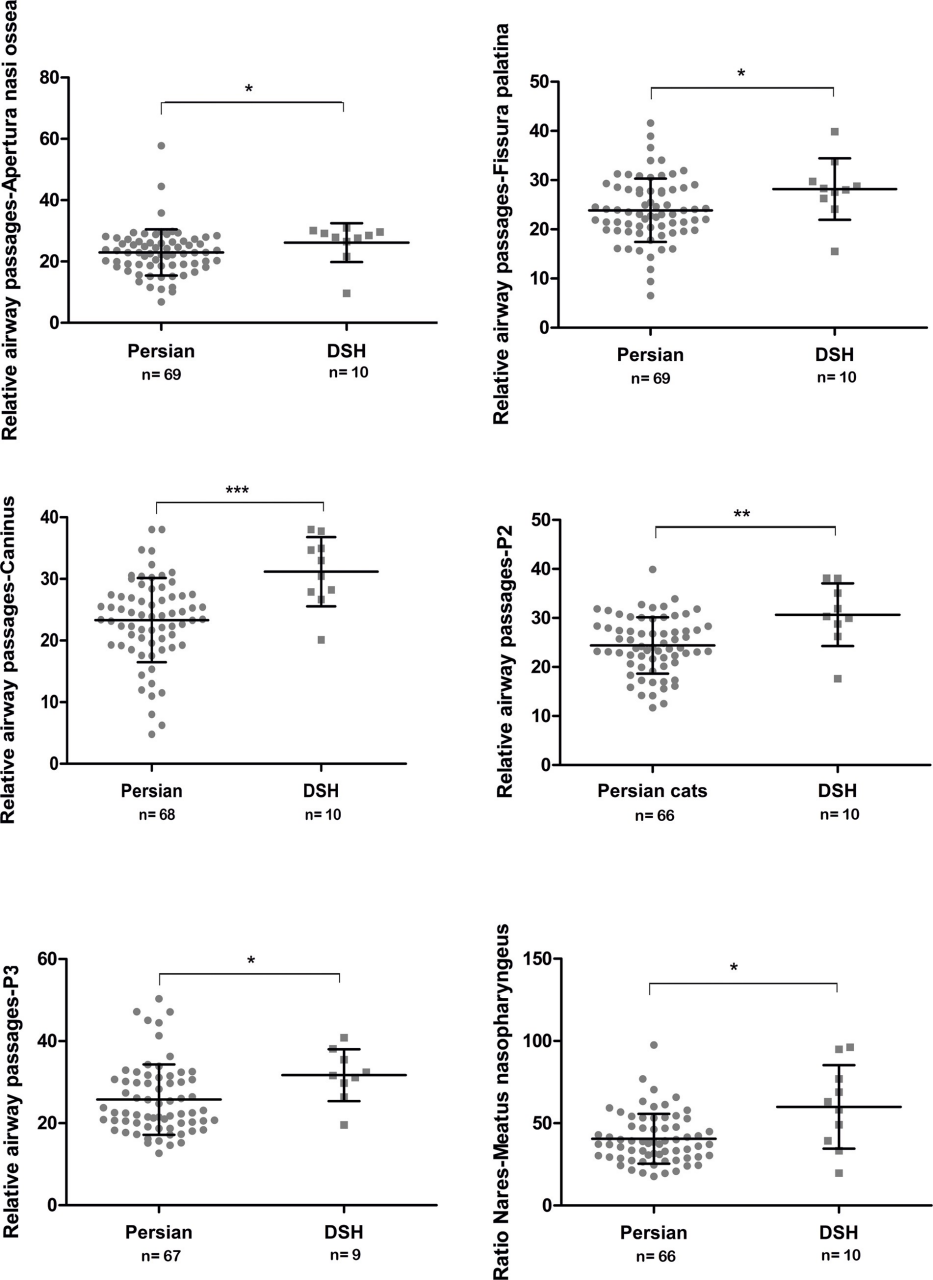
Comparison of the relative airway passages in different cross-sections between Persian and DSH cats. Comparison of the relative nasal airways between Persian and DSH cats at the level of different cross-sections. The values of the individual cats, the mean values, the standard deviations and the significance level are indicated * = p < 0.01; ** = p < 0.001 *** = p < 0.0001.

All measured relative areas of the airways were significantly lower in Persians compared to DSHs (Figs 14 and 15). The measured values are summarised in Table 2. A correlation between the skull, facial and cranial indices, as well as the craniofacial angle and the areas of the airways could not be found in the group of Persian cats. One exception was the smaller nares in relation to the nasopharyngeal meatus, which correlated with an increased facial index (p = 0.037, r = −0.257). Age, sex and body weight were not correlated (p > 0.05). Due to the missing correlation, regression analysis could not be performed.

**Table 2:** Summary of the area comparison of the nasal airways of Persian and Domestic shorthair cats. Area 1/7: area of the nares in relation to area of the meatus nasopharyngeus; area 2: upper canine; area 3: palatine fissure; area 4: bony nasal aperture; area 5: second premolar; area 6: third premolar.

**Breed**	Height of bony nasal aperture	Area 1/7	Area 2	Area 3	Area 4	Area 5	Area 6
**Persian cat**	66.28% ± 4.26%	40.58% ± 15.16%	23.32% ± 6.82%	23.88% ± 6.45%	22.94% ± 7.53	24.40% ± 5.76%	25.76% ± 8.58%
**Domestic shorthair cat**	69.47% ± 1.91%	59.95% ± 25.38%	31.17% ± 5.61%	28.20% ± 6.25%	26.17% ± 6.36%	30.66% ± 6.38%	31.70% ± 6.31%
**p-value**	**p < 0.001**	**p = 0.015**	**p < 0.01**	**p = 0.046**	**p = 0.014**	**p = 0.003**	**p = 0.021**

### Dental malalignment

**Fig 16:**
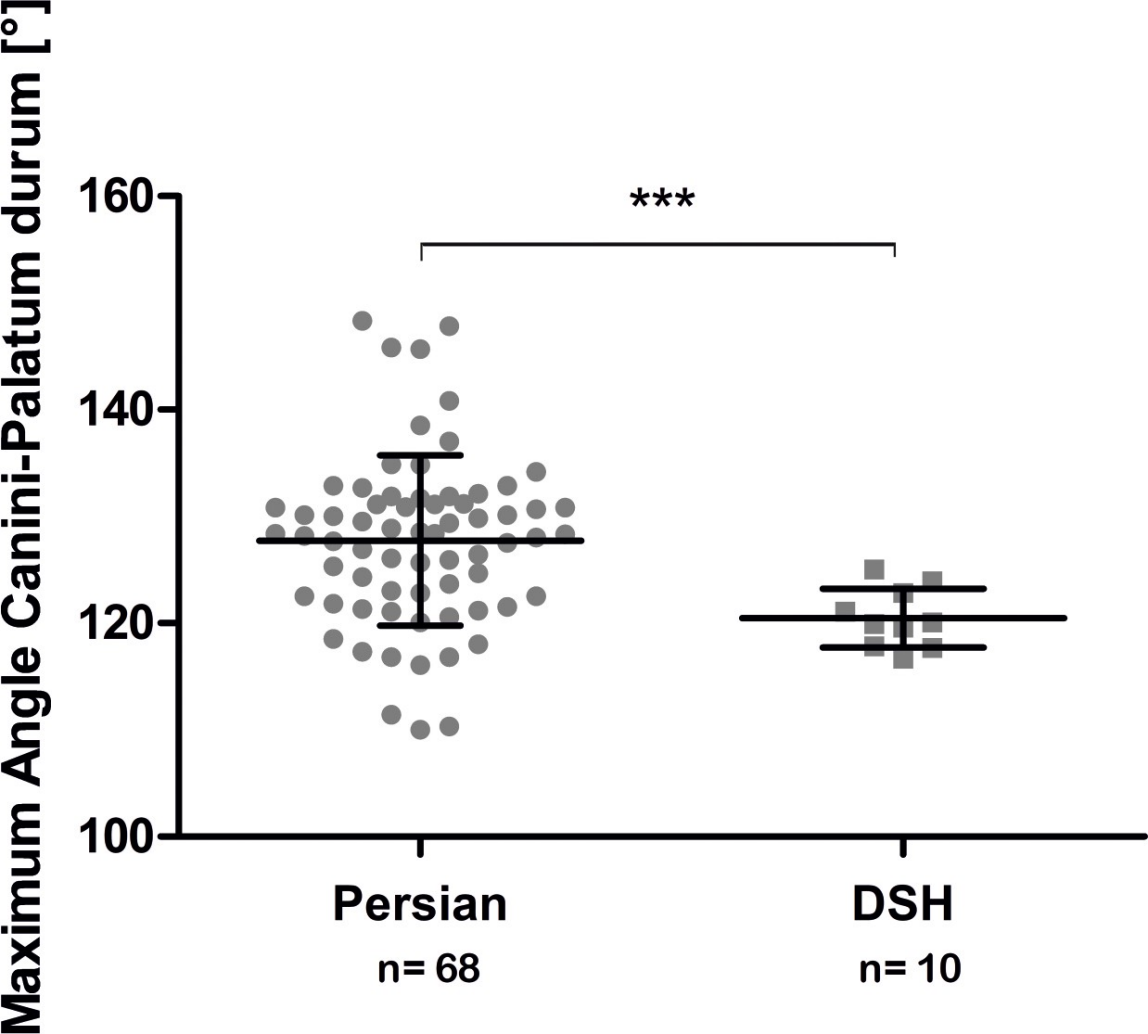
Comparison of the maximum angle between the canine tooth and the hard palate between Persian and Domestic shorthair cats. Graphic representation of the maximum angle between the canine tooth and the hard palate, comparing between Persian and DSH cats. The values of the individual cats, the mean values, the standard deviations and the significance level are indicated. *** = p < 0.0001.

The mean canine angle in Persian cats (127.73° ± 7.97°) was significantly higher (p < 0.001) than the mean canine angle in DSH (120.46° ± 2.76°). There were overlaps between the values of the Persian cats and the DSH (Fig 16), but the angles of the Persian cats (110–148.3°) varied significantly more than the angles of the DSH (116.7–125°). Within the Persian cat population, there was no relevant correlation between the brachycephalic skull dimensions and the canine angle.

The hard palate in Persian cats showed a curved form (p < 0.001) significantly more frequently. The degree of brachycephaly (increase of facial index, cranial index, skull index and decrease of craniofacial angle) and the curved form of the hard palate were significantly positively correlated (p < 0.01 each). The skull index (p < 0.001, F = 13.02) and facial index (p < 0.001, F = 12.42) had the highest effect on the form of the hard palate. In addition, the Persian cats had significantly more tooth malposition in the maxilla (2.03 ± 1.58 teeth) than the DSH (0.11 ± 0.33 teeth; p < 0.001). In the case of the Persian cats, the number of unphysiologically placed teeth varied between 0–6 teeth, whereas in the DSH, either none or only a single tooth had an abnormal position. The most affected tooth was the fourth premolar, which had turned with different intensity around its own longitudinal axis. Often, with the deviating position of the fourth premolar, a simultaneous medial lingual displacement of the first molar was observed. The third maxillary premolar was only occasionally incorrectly positioned.

Within the Persian cat population, there was a significant correlation between craniometric parameters and the number of unphysiologically placed teeth. A larger number of tooth displacements resulted from an increase of facial index (p < 0.001, Spearman’s r = 0.460), cranial index (p < 0.001, Spearman’s r = 0.446) and skull index (p < 0.001, r = 0.495). A decrease of the craniofacial angle, which was associated with a higher degree of brachycephaly, was also responsible for a significantly larger number of tooth displacements (p < 0.029, r = −0.321). The dimensions of the braincase (cranial index; p < 0.01, F = 14.90) and the entire skull (skull index; p < 0.01, F = 13.62) had the most influence on the position of the teeth. There was no influence of age, sex or body weight of the cats.

Similarly, the Persian cats had significantly more tooth displacements in the mandible (1.30 ± 0.99 teeth) than DSH (0.22 ± 0.67 teeth; p = 0.002). Only a quarter of the Persians did not show any deviating tooth positions. Generally, the first molar was affected, occasionally the fourth maxillary premolar and only occasionally the third premolar. In the lower jaw, no statistical correlations could be established between craniometric parameters, gender, age or body weight within the Persian cat population and the number of displaced teeth (p > 0.05).

## Discussion

A brachycephalic phenotype is one potential cause of serious lifelong health problems in domestic animals, which have a severe impact upon their welfare [2–7, 10]. Increased reduction of the facial bones is a conformational risk factor for respiratory compromise and other structural and functional disorders in brachycephalic dogs [27]; however, little research into brachycephaly and its associated disorders has been performed in cats [5]. A correlation between a reduced muzzle length and the risk of developing respiratory compromise was found in a group of brachycephalic cats including variable breeds [5]. In Persians, which have the highest degrees of brachycephaly amongst cat breeds [6, 10], it was never investigated how a reduced longitudinal extension of the skull affects the dimensions of the nasal airways, the morphology of the orbit and physiological dentition. The present study therefore investigates whether the grade of brachycephaly, measured by different cranial indices, is correlated with exophthalmos, dimensions of the nasal passages and dental malalignment in Persian cats.

Although it was previously stated that the impression of flatness of the orbit is a purely optical effect created by the reduction of facial bones [28], the results of this study clearly demonstrated a decreased capacity of the orbital container for the eye globes in Persian cats. We also showed that there is a significant correlation between an increased grade of brachycephaly (cranial index and skull index) and the percentage of the globe that is not enclosed by the bony orbit, which is referred to as exophthalmos [29]. The exophthalmos seen in Persian cats was euphemistically described as ‘physiological’ [11, 12, 30–32], and was defined as one of the desired traits in the modern Persian [4]. However, it was previously shown that exophthalmos results in increased corneal exposure, which is in turn related to decreased corneal sensitivity [33], corneal ulcerations and sequestra [34] in brachycephalic cats [32] and dogs [35–37]. In addition, the highly exposed bulbi promote anatomical entropion and predispose to ocular proptosis [11]. The inclusion of this trait into the breed standard is therefore clearly not recommended from a medical point of view.

The areas of the nasal airways were all significantly smaller in the Persians, however, there was mostly no correlation between these areas and the grade of brachycephaly. Only the relation of the nares to the area of the meatus nasopharyngeus correlated with reduced facial bones. There was no significant difference in the absolute area of the meatus nasopharyngeus, which is why the lower relation of the two areas must be caused by the smaller nares. Stenosis of the nares is also one of the main morphological abnormalities in brachycephalic dogs with BOAS [27]. A large entrance area to the nose is the most important determinant for maximising the breath volume in felids [38]. According to Poiseuille’s law, even minor constrictions in the entrance zone of air flow result in increased resistance and a relevant impairment of breathing capacity, which is why large nares and nasal apertures are important for high-speed runners in felines and other species [39, 40]. Despite the measured difference between DSH and Persians, there was a high overlap between the two groups of cats. While some Persians with clinical signs of respiratory noise and inspiratory effort at rest had very reduced areas of the nares, others had values close to those of DSHs. The true impact of the reduced nares for increased respiratory difficulty therefore remains unclear. The study population was heterogenous with respect to age (4 months and 16 years) and some of the clinical signs resulting from the brachycephalic head conformation have a rather chronic course and could potentially be present at a later time. This may have negatively biased the results concerning the relation between morphological and clinical findings. It was also interesting to note that most breeders mentioned keeping a large group (up to 20) of cats, but they only introduced three or four cats of their breeding stock. Owner preselection towards clinically normal cats may have also biased a clear correlation between clinically evident respiratory noise and increased brachycephaly. A correlation of brachycephalic skull features with respiration after exercise would have been ideal in a prospective study and was, in fact, initially aimed for, but was, in the end, not feasible due to missing compliance of cats. Computational flow dynamic simulation of nasal airflow may overcome this problem and allow for determination of a correlation between morphological and physiological features of the feline respiratory system [41].

The lack of correlation between the other areas of the airways and the grade of brachycephaly seems somewhat implausible or at least unexpected, as it was previously shown that a shorter muzzle results in increased respiratory compromise in cats [5]. It seems plausible that the dimensions of the nasal airways depend on the proportions of the entire nasal cavity, which in turn is largely determined by dimensions of the nasal, palatal and maxillary bones. One subjective finding in the CT images was a massive reduction of the frontal sinuses in most of the Persian cats. In more than a third, they were totally absent or reduced to a minimum (Fig 2). It could be possible that the reduction of facial bones affects the ventral airflow passages much less, but rather the dorsally located frontal sinuses. A potential limitation to consider is the assumption that millimetre and submillimetre differences in the nasal airways cannot be reliably measured using graphical software and a missing correlation was caused by inaccurate measurements.

It has already been described in the literature that shortening of the jaw in brachycephalic cats, especially in the extreme brachycephalic Persian cat, leads to a crowding and impaction of the molar teeth [3]. Our investigation proved this finding and revealed that shortening of the facial bones (high facial index) and the neurocranium (high skull index) are correlated with dental malalignment in the upper but not the lower jaw. The lack of space in the upper jaw forces individual teeth to rotate in a transverse position or to relocate to the buccal or lingual side of the other teeth [6]. The most affected tooth was the fourth maxillary premolar, which is an important carnassial tooth in the cat [42]. The more or less normal tooth position in the lower jaw further impairs the functional relationship between upper and lower teeth. It is therefore likely that mastication is impaired in extremely brachycephalic Persian cats. This malocclusion also promotes retention of food in the oral cavity, and brachycephalic cats are predisposed to dental plaque formation and periodontitis [7], which has also been found in brachycephalic dogs [43]. The clinical finding of gingivitis or periodontal ulcers resulting from malocclusion were not correlated with increased brachycephaly. Although the development of periodontal disease is promoted by malocclusion in brachycephalic breeds [44], it is a chronic, multifactorial disease and influenced by a number of factors such as diet, bacterial flora, nutritional deficiencies of vitamins [45], as well as genetic predispositions [46]. Again, owner preselection of cats made available for the study may have biased the results.

Although brachycephalic cats are frequently affected by rotated maxillary canines [6], there was no correlation between the degree of brachycephaly and the angle of the canine teeth to the hard palate within the Persian cat population. A longitudinal reduction of the maxilla can cause a caudal shift of the alveolar space, which applies mechanical force on the canine teeth that rest on the teeth of the lower jaw. This can result in a near horizontal position of the canines [6]. The missing correlation of canine angulation and increased brachycephaly is therefore striking. Brachycephaly in animals is not a single trait, but rather seems to be a morphological feature of different diseases. A premature closure of cranial base synchondroses [47] and facial sutures has been found in brachycephalic dogs [48]. A craniosynostosis of the coronal sutures was found in some Persian cats that affects longitudinal neurocranial growth and reduces cranial capacity [10], which are two completely different disease entities. The orbital flatness found in the present study could be explained by the compensatory growth of the neurocranium into the orbit, which occurs perpendicular to the coronal sutures [10]. The impacts on the facial bones in such a craniosynostosis is rather unknown in cats. Although the depressed bridge of the nose and depressed middle of the face were clearly present, this ‘midface hypoplasia’ appears, in general, not to be caused by a reduction of the facial bones but at least partly by the prominent forehead that projects rostrally over the nose of the cats (frontal bossing). It seems that the nasal passages are at least partially repositioned ventrally underneath the neurocranium and achieve an oblique position (Fig 2), as found in another study [6]. It is possible that the maxillary and palatine bones are distorted rather than absolutely shortened. However, this remains speculative without further information on physiological and pathological skull growth in cats. A fundamental analysis of the growth trajectories of Persian skulls during ontogeny would be necessary to determine the exact impact of impaired neurocranial growth on facial growth and conformation in cats.

## Conclusion

There is a clear correlation between increased grades of brachycephaly and exophthalmos, stenotic nares and dislocation of the fourth maxillary premolar tooth in Persian cats. The phenotypic variability within the breed of Persian cats is high and craniometric parameters can overlap between DSH and Persians, which potentially allows ‘outbreeding’ of extreme brachycephaly and the reduction of its negative effects on health without the introduction of other cat breeds.
